# Acute night shift work is associated with increased blood pressure and reduced sleep duration in healthy adults

**DOI:** 10.14814/phy2.70231

**Published:** 2025-02-12

**Authors:** Sophie L. Seward, Erin E. Kishman, Corey A. Rynders, Josiane L. Broussard

**Affiliations:** ^1^ Colorado State University Fort Collins Colorado USA

**Keywords:** ambulatory blood pressure, blood pressure dipping, cardiovascular disease, free‐living, shift work

## Abstract

Shift workers have a 40% higher risk for cardiovascular disease (CVD) compared to people who work day shifts. However, the acute impact of shift work on CVD risk factors in free‐living settings remains unclear. We therefore investigated the impact of acute night shift work on factors related to cardiovascular health including blood pressure (BP) and sleep duration. Twenty‐four rotating shift workers (19F, 23 ± 4 y, BMI: 23 ± 3 kg/m^2^; mean ± SD) participated in a quasi‐randomized crossover study. Assessments were conducted over the course of 1 day shift and one night shift in a free‐living setting. BP was measured every 30 min by an ambulatory monitor. Sleep and wake times were recorded. Mixed effects models were conducted to examine changes in variables between conditions. Acute night shift work was associated with significantly higher 24 h systolic (107 ± 1 vs. 104 ± 1 mmHg; *p* < 0.0001) and diastolic (67 ± 1 vs. 64 ± 1 mmHg; *p* < 0.0001) BP, as well as blunted dipping patterns in systolic BP (8 ± 1 vs. 12 ± 1%; *p* = 0.032), as compared to day shift work. Sleep duration was significantly shorter during the night shift as compared to the day shift (4 h 04 ± 19 min vs. 8 h 22 ± 18 min; *p* < 0.0001). As little as one night of shift work in a free‐living setting is sufficient to induce multiple CVD risk factors including increased BP and reduced sleep duration in healthy adults. It is critical to identify strategies to prevent or attenuate the negative impact of shift work on CVD risk in a large portion of the working population.

## 1 INTRODUCTION

Over one quarter of the US workforce engages in evening, night, or rotating shifts (i.e., shift work) to meet the requirements of modern society (Torpey, 2015). People who conduct shift work have a 40% higher risk for developing cardiovascular disease (CVD) compared to people who work day shifts (Bøggild & Knutsson, 1999; Kawachi et al., 1995; Tenkanen et al., 1997; Vetter et al., 2016). Furthermore, the duration of exposure to shift work is related to CVD risk. For example, in a prospective study in female nurses with varying years of shift work history, nurses who conducted 10 years of shift work were twice as likely to develop CVD compared to nurses who spent <5 years performing shift work (Vetter et al., 2016). Although age is a leading risk factor for CVD, rates of CVD in middle‐aged and older adults (>50 years) have been slowing while CVD rates in adults aged 18–50 years have been steady or increasing (Andersson & Vasan, 2018), highlighting the need to examine CVD risk factors in young adults.

Aside from age, elevated blood pressure (BP) is the leading risk factor for CVD (Kovell et al., 2015). Furthermore, BP alterations at specific times of the day are independently associated with elevated CVD risk. For example, during a normal night of sleep in healthy adults, there is a 10%–20% dip in BP compared to waking values (referred to as “BP dipping”). Blunting of this BP dip (defined as a < 10% reduction from pre‐sleep to sleeping BP) is indicative of elevated CVD risk even in normotensive individuals. Furthermore, in healthy adults, there is a 10–20 mmHg increase in BP within the first 2 h of waking as compared to the final 2 h of sleep, typically referred to as the morning BP surge (Booth III et al., 2020). An exaggerated morning BP surge (defined as >20 mmHg SBP) has also been associated with an increased risk for CVD (Kario et al., 2003; Li et al., 2010; Metoki et al., 2006). Together, these findings highlight the importance of assessing BP profiles over the entire 24 h period to uncover important and clinically relevant changes in BP across the day and night during both wake and sleep.

Findings from controlled in‐laboratory studies in healthy adults with no prior history of shift work reveal that just two nights of simulated night shift work using a highly controlled inpatient protocol induces increases mean 24 h BP as well as blunted BP dipping (Morris et al., 2016). Consistent with these findings, results from another controlled inpatient study in chronic shift workers found that simulated night shift work increases BP even in people with a history of shift work (Morris et al., 2017) highlighting elevated BP and loss of BP dipping as a potential mechanisms by which shift work contributes to the risk of CVD in this population. However, the impact of shift work on the waking BP surge was not elucidated.

Despite the finding that simulated night shift work in healthy adults acutely increases BP and blunts BP dipping in controlled inpatient settings, studies on the impact of shift work on BP patterns in a free‐living environment are limited. Results from one outpatient study examining 12‐h shifts, showed BP to be elevated during the night shift when compared to the day shift (Su et al., 2008). However, BP dipping or waking BP surge were not examined. Thus, there is a critical need for studies that examine the acute effects of night shift work on BP patterns in a controlled yet ecologically valid free‐living environment.

In another outpatient study, participants who conducted rotating shift work had higher 24 h BP as well as blunted BP dipping when they performed night shift work as compared to a nonwork day (Patterson et al., 2021). This study was conducted in emergency medical services workers, in whom job stress and BP are presumably higher during work days as compared to nonwork days, regardless of whether the shift occurred at night. Furthermore, there was a large range of age and body mass index (BMI) in this study, which can have significant impacts on BP. As such, the heterogeneity of populations in many earlier studies, including wide ranges in age, BMI, and job stress levels, has limited the ability to draw precise conclusions about the direct impact of night shift work on BP.

Results from another study conducted in young, healthy medical residents demonstrated that BP was higher during a 24 h shift compared to an 8 h day shift (Fialho et al., 2006). Finally, in young, healthy female nurses, evening and night shift work was associated with higher 24 h BP and sleep BP as compared to day work (Lo et al., 2008). However, in the aforementioned studies, information on shift work history was not provided, therefore, the timeline of BP impairments induced by shift work exposure remains unclear.

To assess the timeline of BP impairments associated with shift work exposure, one study examined 24 h ambulatory BP in newly‐hired transit workers in a free‐living setting. After 6 months of shift work participants experienced a significant blunting of sleep BP dipping as compared to baseline values (McHill et al., 2022). However, there were no restrictions to age or BMI in this study, thus the population was highly heterogenous.

Taken together, findings from research on CVD risk factors in free‐living settings suggests that shift work is consistently associated with significant increases in BP and blunted BP dipping. While valuable, prior research has not simultaneously assessed key parameters such as BP dipping and waking BP surge, and nearly all prior free‐living studies have been conducted in heterogenous populations with varying history of shift work exposure and varying degrees of job stress. Thus, disentangling the impact of night shift work on BP patterns in free‐living individuals has proven challenging.

Our study addresses many of these gaps by focusing on a homogeneous group of young, healthy adults with limited exposure to shift work, providing a clearer understanding of how acute night shift work influences BP patterns and associated cardiovascular risk factors in free‐living conditions. We hypothesized that a single night shift would be associated with increases in CVD risk factors such as increased 24 h BP and blunted BP dipping compared to day shift work.

## 2 MATERIALS AND METHODS

### 2.1 Participants

Healthy adults 18–35 years old and with a BMI of 18.5–29.9 kg/m^2^ were recruited for this study. Participants were intermittent rotating shift workers (<2 years of rotating shift work exposure), defined as working at least 1 day shift and one night shift per month. Participants were excluded if they had any clinically significant medical or surgical conditions within the last year; were diagnosed with diabetes or CVD, taking hypertensive medication, or pregnant as determined by urine hCG test. All participants provided written informed consent to participate, and all procedures were reviewed and approved by the Colorado State University Institutional Review Board.

### 2.2 Experimental protocol

**FIGURE 1:**
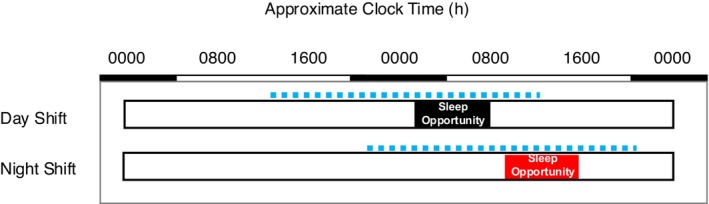
Overview of experimental protocol. Data collection was conducted in a quasi‐randomized crossover order under free‐living conditions for two work shifts including a day shift (top; a) and a night shift (bottom; b). SBP, DBP, and HR were recorded every 30 min as represented by the blue dotted line. The gray rectangle indicates the mean self‐reported sleep period during day shift ± SEM. The red rectangle indicates the mean self‐reported sleep period during night shift ± SEM.

Participants underwent a quasi‐randomized crossover study conducted under two free‐living conditions consisting of approximately 24 h starting approximately 20 h prior to wake time and ending approximately 4 h after wake. An example study protocol is shown in Figure 1. A quasi‐randomized design was used because the order of conditions often depended on participant work schedules rather than true randomization. Day shift and night shift conditions were conducted approximately 3 months apart (110 ± 25 d). The day shift was defined as a shift that commenced at or before 09:00. The night shift was defined as a shift that commenced at or after 19:00. Total sleep duration, sleep onset, and sleep offset (i.e., wake time) were recorded and verified by sleep logs and call‐ins or texts to a time stamped recorder.

### 2.3 Blood pressure monitoring

Blood pressure assessments were collected every 30 min throughout the study with an ambulatory OnTrak 90,227 monitor (Spacelabs™, Snoqualmie, WA) to assess systolic BP (SBP), diastolic BP (DBP), and heart rate (HR). Participants were instructed to remain still during ambulatory BP measurements to reduce monitor errors and ensure accurate readings. In the event of an error message, the ambulatory BP monitor repeated the measurement approximately 2 min later. Participants were instructed to refrain from wearing the ambulatory BP monitor while driving, showering, or exercising to mitigate safety concerns and potential monitor errors. Participants were instructed to discontinue the use of the ambulatory BP monitor if the cuff induced discomfort to the extent that it hindered their ability to sleep. Participants were instructed to resume ambulatory BP monitoring upon wake if they removed it during sleep. Prior to analyses, the ambulatory BP data underwent visual inspection to ensure completeness.

The expected number of ambulatory BP observations were calculated by multiplying the total wear time by 2, given that BP measurements were taken every 30 min. The actual number of ambulatory BP observations were recorded. The ratio of actual observations to expected observations were calculated and expressed as a percentage. Further, total ambulatory BP monitor wear time was calculated as the duration between the first record of an BP measurement and the last record of BP measurement in each condition. To minimize discomfort, the ambulatory BP monitor automatically inflated to 130 mmHg; however, if SBP was not detected, the cuff automatically increased pressure in increments of 30 mmHg until SBP was detected. Participants were blinded to the ambulatory BP results during the study.

### 2.4 Blood pressure parameters

Twenty‐four‐hour BP was defined as the mean BP during the entire collection period. Wake and sleep BP were defined as the mean BP during self‐reported wake or sleep periods based on sleep logs and call‐ins. Blood pressure dipping was calculated as the percentage change from mean waking BP to mean sleeping BP. The BP dipping calculation has been previously described (O'Brien et al., 2013). Different definitions and thresholds have been used to define the blood pressure surge that occurs after waking up calculated by subtracting the lowest sleeping blood pressure from the waking blood pressure (Booth III et al., 2020). Most reports use the phrase “morning BP surge”, however, in participants who perform shift work, “morning” is likely not the same as “waking” and therefore may not be appropriate. For our purposes, we use the term “waking surge” calculated as the absolute difference between the mean BP from the 2 h before to the 2 h after waking up. This has also been referred to as the “prewaking surge” in some studies (Booth III et al., 2020).

### 2.5 Statistical analyses

Statistical analyses were conducted using SAS (version 9.4; Cary, NC) with a significance threshold of *α* = 0.05. To examine differences between SBP, DBP, and HR during the 24 h period, wake periods, and sleep periods, mixed‐effect linear models with a random intercept for participant ID were used. Mixed‐effects linear models were performed as this method utilizes maximum likelihood estimation which allows for the use of all available data. The differences between BP dipping, waking BP surge, sleep duration, sleep onset, and sleep offset were also examined between day shift and night shift using a mixed‐effects linear model. Mixed‐effects linear models were performed to use all available data. Spearman's correlations were calculated as a separate exploratory analysis to examine potential relationships between changes in sleep duration and changes in the blood pressure dip and surge outcomes.

Time since wake was determined based on self‐reported wake time. BP and HR were aggregated into 30‐min intervals based on time since wake. Mixed‐effect linear models with a condition by time (time since wake) interaction were used to examine whether BP and HR were different over the 24 h period between night shift and day shift conditions. SBP, DBP, and HR were dependent variables. All models were adjusted for ambulatory BP monitor wear time. Results are presented as least‐squares mean ± standard error of the mean (SEM), except for baseline characteristic data, which are displayed as raw mean ± standard deviation (SD).

## 3 RESULTS

### 3.1 Participants

A total of 27 adults were assessed for eligibility, 25 were enrolled, and one participant dropped out of the study due to arm numbness due to the ambulatory BP monitor. Twenty‐four participants completed at least one condition of the study (23 ± 4 y; 23 ± 3 kg/m^2^, mean ± SD; Table 1). Of those 24, 83% were female (*n* = 20), 8% Asian (n = 2), 4% American Indian (*n* = 1), 4% Black/African American (*n* = 1), 75% White (*n* = 18), and 8% two or more races (*n* = 2). Twenty participants completed both conditions. Participants had performed intermittent night shift work for 173 ± 32 days (mean ± SEM); range 0–491 days; at least one night shift per month. In this quasi‐randomized study, 16 participants completed the day shift condition first whereas eight participants completed the night shift condition first.

**TABLE 1:** Baseline participant characteristics.

*n*	24
Female	19
Age, y	23 ± 4
Weight, kg	66 ± 11
Height, cm	167 ± 9
BMI, kg/m^2^	23 ± 3

*Note*: Data are presented as mean ± SD.

Abbreviation: BMI, body mass index.

Over 90% of scheduled BP measurements (every 30 min) were successfully collected across both conditions. Since data collection ended shortly after waking up, the total wear time for the ambulatory BP monitor was approximately 2.5 h shorter during night shift work as compared to day shift work (15 h 28 ± 49 min vs. 18 h 6 ± 45 min; *p* = 0.026).

### 3.2 Shift work and systolic and diastolic blood pressure

Night shift work was associated with significantly higher mean 24 h SBP (night: 107 ± 1 mmHg vs. day: 104 ± 1 mmHg; *p* < 0.0001) and DBP (night: 67 ± 1 mmHg vs. day: 64 ± 1 mmHg; *p* < 0.0001) as compared to day shift work (*p* < 0.0001 for both; Table 2). There were no differences in mean waking SBP between conditions (Table 2). However, mean sleeping SBP was higher during night shift work as compared to day shift work (night: 100 ± 2 mmHg vs. day: 96 ± 1 mmHg; *p* = 0.0004; Table 2). There were no differences in waking or sleeping DBP between conditions (Table 2).

**TABLE 2:** BP and sleep characteristics.

	Day shift (*n* = 24)	Night shift (*n* = 20)	*p* Value
24 h SBP (mmHg)	104 ± 1	107 ± 1	<0.0001*
24 h DBP (mmHg)	64 ± 1	67 ± 1	<0.0001*
24 h HR (mmHg)	68 ± 2	67 ± 2	0.156
Waking SBP (mmHg)	110 ± 1	109 ± 1	0.481
Waking DBP (mmHg)	69 ± 1	70 ± 1	0.073
Waking HR (bpm)	73 ± 2	69 ± 2	<0.0001*
Sleeping SBP (mmHg)	96 ± 1	100 ± 2	0.0004*
Sleeping DBP (mmHg)	56 ± 1	56 ± 1	0.800
Sleeping HR (bpm)	62 ± 2	62 ± 2	0.499
Sleeping SBP dip (%)	12 ± 1	8 ± 1	0.032*
Sleeping DBP dip (%)	19 ± 1	19 ± 2	0.888
Waking SBP surge (mmHg)	12 ± 1	6 ± 2	0.013*
Waking DBP surge (mmHg)	11 ± 1	8 ± 1	0.194
Sleep duration (h:min)	8:22 ± 0:18	4:04 ± 0:19	<0.0001*
Sleep time onset (h:min)	22:55 ± 0:14	7:16 ± 0:15	<0.0001*
Sleep time offset (h:min)	7:17 ± 0:20	11:23 ± 0:21	<0.0001*

*Note*: Data are presented as least square mean ± SEM. Least square means were analyzed by a linear mixed model.

Abbreviations: bpm, beats per minute; DBP, diastolic blood pressure; h, hour; HR, heart rate; SBP, systolic blood pressure.

* *p* Value ≤0.05.

**FIGURE 2:**
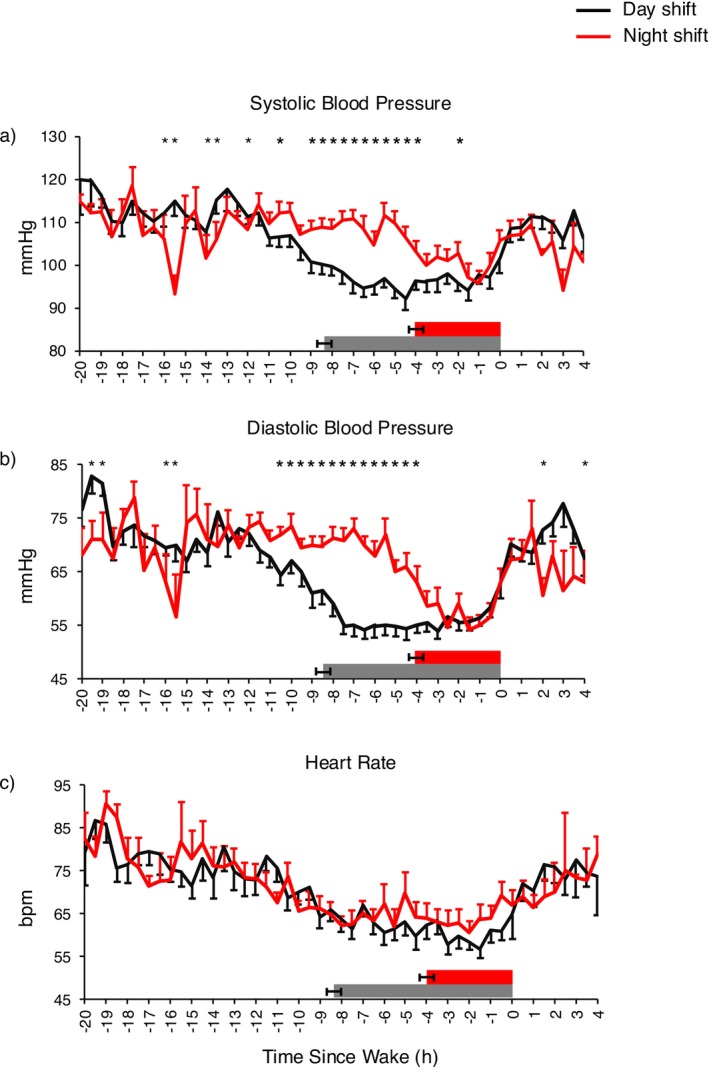
Systolic blood pressure (left; a), diastolic blood pressure (middle; b) and heart rate (right; c) readings assessed every 30 min during each condition and analyzed by mixed‐effects model. Data are presented as least square means ± SEM. The gray rectangle indicates the mean self‐reported sleep period during day shift ± SEM. The red rectangle indicates the mean self‐reported sleep period during night shift ± SEM. *p value <0.05.

There was a significant condition by time interaction for 24 h SBP and DBP (*p* < 0.0001 for both), indicating that BP profiles were different between night shift work and day shift work. SBP and DBP were significantly different at multiple timepoints between conditions (Figure 2a,b).

### 3.3 Shift work and heart rate

Twenty‐four‐hour heart rate (HR) was not different between conditions (Table 2). In contrast, waking HR was lower during night shift work compared to day shift work (night: 69 ± 2 bpm vs. day: 73 ± 2 bpm; *p* < 0.0001; Table 2). Sleeping HR was not different between conditions (Table 2). Finally, HR profiles were not different between conditions (*p* = 0.183; Figure 2c).

### 3.4 Shift work and blood pressure dipping

**FIGURE 3:**
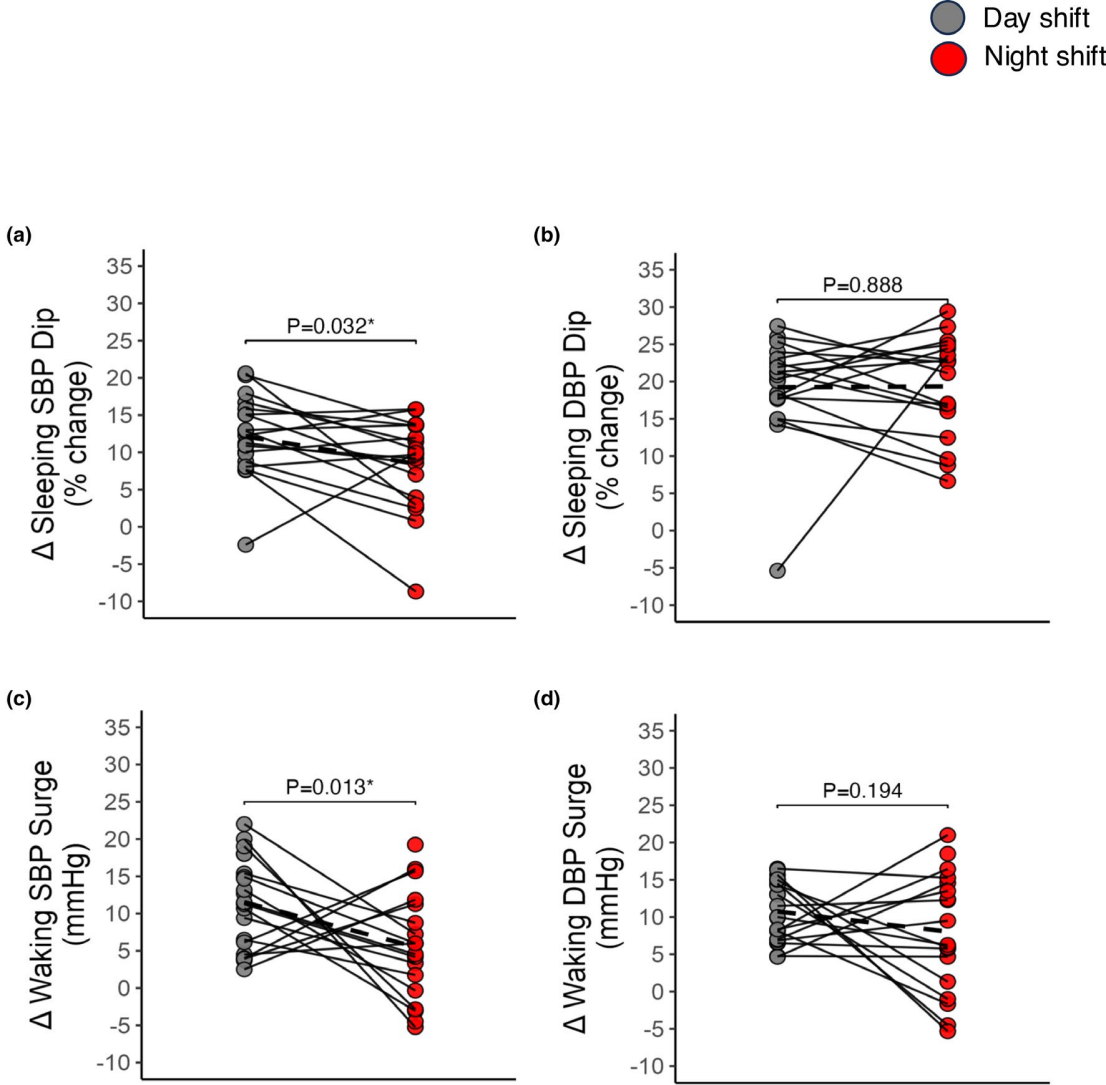
Individual blood pressure dip (a and b) and surge (c and d) responses to day shift versus night shift work. Blood pressure dip was calculated as the percent change from mean waking to mean sleeping blood pressure. Blood pressure surge was calculated as the absolute difference between the mean blood pressure from the 2 h before to the 2 h after waking up. Individual responses indicated by the solid lines; mean responses for each outcome are indicated by the dashed lines. Data were analyzed using mixed‐effects models. *p value <0.05.

Night shift work was associated with a significantly blunted SBP dip during sleep as compared to day shift work (night: 8 ± 1% vs. day: 12 ± 1%; *p* < 0.0001; Table 2, Figure 3a), which is below the clinical cut off for blunted dipping associated with increased CVD risk. There were no differences in DBP dipping between conditions (Figure 3b).

Night shift work was associated with a smaller waking SBP surge compared to day shift work (night: 6 ± 2 mmHg vs. day: 12 ± 1 mmHg *p* = 0.013; Table 2, Figure 3c). In contrast, there were no differences in the waking DBP surge between conditions (Table 2, Figure 3d).

### 3.5 Shift work and sleep duration and timing

Night shift work was associated with a significantly shorter sleep duration as compared to day shift work (night: 4 h 04 ± 19 min vs. day: 8 h 22 ± 18 min; *p* < 0.0001; Table 2), as well as significantly later sleep onset (night: 7:16 a ± 15 min vs. day: 10:55 p ± 14 min; *p* < 0.0001) and sleep offset (night: 11:23 a ± 21 min vs. day: 7:17 a ± 20 min; *p* < 0.0001; Table 2). Spearman's rank correlations between the change in sleep duration (Δ = night shift–day shift) and the blood pressure outcomes were not significant (Spearman's Rho for BP outcome vs. Δ sleep duration: ΔSBP dip, ρ = −0.09, *p* = 0.76; ΔDBP dip, ρ = −0.004, *p* = 0.99; ΔSBP surge, ρ = 0.11, *p* = 0.70; ΔDBP surge, ρ = −0.38, *p* = 0.16).

## 4 DISCUSSION

The purpose of this study was to examine the impact of acute night shift work on CVD risk factors in otherwise healthy participants with a relatively short history of intermittent night shift work exposure. We found that acute night shift work was associated with increased mean 24 h SBP and DBP, as well as blunted SBP dipping as compared to day shift work. Further, we found that acute night shift work was associated with a significant reduction of over 4 h in self‐reported total sleep duration compared to day shift work. These findings in a homogenous and otherwise healthy population are consistent with previous studies and support the idea that acute night shift work is associated with increased CVD risk.

Night shift work was associated with a 3 mmHg increase in mean 24 h SBP and DBP compared to day shift work. These results are consistent with a 4 mmHg increase in both SBP and DBP found in young, healthy medical residents during a 24 h work shift as compared to a 8 h day shift work (Fialho et al., 2006), and a 1 mmHg increase in both SBP and DBP during simulated night shift work compared to a simulated day shift work in chronic shift workers (Morris et al., 2017). Despite an increase, SBP and DBP remained within the normal range (<130/80 mmHg). However, results from prior research indicate that progressive increases in BP increase the risk of CVD even in healthy, normotensive adults (Whelton et al., 2020). Therefore, the increase in BP induced by acute night shift work in this study represents a clinically significant increase and supports the link between chronic night shift work and CVD risk (Bøggild & Knutsson, 1999; Kawachi et al., 1995; Tenkanen et al., 1997; Vetter et al., 2016).

We also demonstrated that acute night shift work blunts SBP dipping as compared to day shift work. Notably, we observed an 8% SBP dip during night shift work compared to 12% during day shift work. Results from epidemiological studies suggest that a BP dip of <10% is associated with future CVD development (Hoshide et al., 2003; Viera et al., 2012), highlighting the clinical relevance of BP alterations induced by night shift work. Our results of blunted BP dipping are consistent with some (Lo et al., 2008; McHill et al., 2022; Morris et al., 2016), but not all (Baumgart et al., 1989; Fialho et al., 2006; Stieler et al., 2019), prior studies of shift work. Methodological discrepancies, such as the differences in total sleep duration or potential napping during night shift work, may explain some of these inconsistencies. For example, in one outpatient study conducted in emergency medical services workers, participants demonstrated blunted BP dipping during a night shift as compared to a nonwork day (Patterson et al., 2021). However, in a subset of participants in this study who napped for >60 min during the night shift, BP dipping was in the normal range of 10%–20% (Patterson et al., 2021), suggesting that insufficient sleep loss is a likely mechanism underpinning blunted BP dipping in night shift workers.

We also observed a significantly lower waking BP surge during night shift work as compared to day shift work. To our knowledge, this is the first study to report the waking BP surge during simulated or free‐living night shift work. A reduction in waking BP surge is contrary to our original hypothesis though not unexpected based on the blunted BP dip, which may limit waking BP surge potential. Previous studies that have demonstrated a link between higher waking BP surge and increased risk of CVD and stroke were conducted in people who slept at night and woke up in the morning (Kario et al., 2003; Li et al., 2010; Metoki et al., 2006). In a previous longitudinal study, however, blunted BP dipping pattern was associated with a smaller morning BP surge and heightened CVD risk (Verdecchia et al., 2012). Thus, the link between elevated waking BP surge and increased CVD likely depends on the population and whether a robust BP dipping pattern is present.

In our hands, acute night shift work was also associated with a 4 h reduction in self‐reported sleep duration, which is consistent with data from previous studies that report short sleep duration during night shift work as compared to day or afternoon shift work (Fialho et al., 2006; Torsvall et al., 1989). Insufficient sleep, defined as less than 7 h of time in bed per night, is independently associated with increased 24 h BP and CVD risk (Ayas et al., 2003; Buxton & Marcelli, 2010; Shankar et al., 2010; St‐Onge et al., 2020; van Leeuwen et al., 2009). For example, results from a randomized crossover study conducted in women showed that insufficient sleep (achieve by reducing sleep by 1.5 h per night) for 6 weeks was associated with increased 24 h SBP compared to habitual sleep (St‐Onge et al., 2020). Furthermore, results from another study in healthy adults demonstrated that participants who were exposed to repeated bouts of insufficient sleep (4 h of sleep/night for three nights followed by recovery sleep of 8 h, repeated four times in succession) had blunted BP dipping and increased 24 h BP as compared to participants who were provided sufficient sleep (Yang et al., 2017). Finally, previous research indicates that for every hour of sleep less than 7 h per night, the risk of coronary heart disease increases by 11% (Wang et al., 2016). Altogether these results suggest that insufficient sleep is likely an important mechanism by which night shift work increases BP and the risk for CVD.

## 5 LIMITATIONS

This study also has several limitations. First, we did not restricted exercise or caffeine in our study, both of which could impact BP and sleep. Second, frequent BP assessments associated with ambulatory monitoring has been associated with discomfort as well as sleep disruptions (Beltman et al., 1996; Henskens et al., 2011; van der Steen et al., 2005). Although BP assessments were conducted in both conditions, they may be more disruptive to sleep during the night shift condition in which sleep occurred later in the morning at a time that sleep is already reported to be disrupted (Åkerstedt, 1988; Knott et al., 2020). Third, we assessed sleep duration by self‐report and did not assess sleep objectively. However, previous studies have demonstrated a strong correlation between self‐reported sleep duration and sleep duration assessed by actigraphy (Hauri & Wisbey, 1992; Lockley et al., 1999). Fourth, our study design was quasi‐randomized due to work schedules that dictated the order of conditions and therefore order effect may have implications on the findings of this study findings. Additionally, our study included young, healthy participants and therefore results may not be generalizable to all shift working populations. We also did not measure physical activity which may or may not be different between day and night shift workers (Lauren et al., 2020; Mansouri et al., 2022) as well as differ between day and night shift in the same individual. Finally, participants were primarily female who were premenopausal and therefore fluctuations in hormones that may impact BP such as estrogen may contribute to our findings.

## 6 FUTURE DIRECTIONS

Night shift work is largely unavoidable in modern society, and the risk for CVD increases with increased exposure to shift work. Thus, the identification, development, and testing of strategies to improve cardiometabolic health and subsequently lower CVD risk in a sizeable portion of the population are warranted. Countermeasures to attenuate the negative effects of shift work may include interventions known to improve cardiometabolic health in other contexts. Indeed, increasing sleep duration may be a viable countermeasure to reduce BP in night shift workers, given that napping for as little as 1 h during night shift work has been shown to restore BP dipping patterns (Patterson et al., 2021). Furthermore, sleep extension by 1 h per night for 6 weeks in participants with elevated BP and who chronically slept less than 7 h was associated with reduced SBP and DBP as compared to baseline levels (Haack et al., 2013). While our results did not reveal a relationship between sleep duration and BP responses during shift work per se, future investigations should attempt to control for sleep duration and/or match sleep duration across conditions. Finally, valuable insights may be derived from studies that combine rigorous, highly controlled inpatient studies of physiological outcomes with free‐living measures of behavior to better understand sources of response variability while maintaining an acceptable level of external validity.

## AUTHOR CONTRIBUTIONS

SLS and JLB developed the idea and designed the study. SLS, EEK, CAR, and JLB analyzed and interpreted the data and wrote the manuscript. All authors were involved in writing the paper and had final approval of the submitted and published versions. JLB is the guarantor of this work and, as such, had full access to all the data in the study and takes responsibility for the integrity of the data and the accuracy of the data analysis.

## FUNDING INFORMATION

This research was supported in part by F31HL165883 to SLS, T32HL149646 to EEK, and R01DK125653 and R01HL168081 to JLB.

## CONFLICT OF INTEREST STATEMENT

No conflict of interest to declare.

## ETHICS STATEMENT

Study participants provided written informed consent to participate. All procedures were reviewed and approved by the Colorado State University Institutional Review Board and were conducted according to the ethical principles of the National Commission for the Protection of Human Subjects of Biomedical and Behavioral Research report entitled Ethical Principles and Guidelines for the Protection of Human Subjects of Research.
